# Predictive performance of ISS and NISS for clinical outcomes in severely injured trauma patients: a retrospective registry study

**DOI:** 10.1007/s00068-026-03198-1

**Published:** 2026-04-28

**Authors:** Aleksi Säkkinen, Nikke Partio, Ville Mattila, Heikki Mäenpää, Antti Riuttanen

**Affiliations:** 1 Tampere University Hospital, Elämänaukio 2, Tampere, 33520 Finland; 2 Faculty of Medicine and Health Technology, Tampere University, Arvo Ylpön katu 34, Tampere, 33520 Finland

**Keywords:** Severe trauma, Injury severity score, New injury severity score, Resource utilization

## Abstract

**Background:**

The Injury Severity Score (ISS) and New Injury Severity Score (NISS) are widely used in evaluation of injury severity in trauma patients. The aim of this study is to assess the predictive accuracy of these scoring systems on clinical outcomes.

**Methods:**

We conducted a retrospective registry study of 1,112 severely injured trauma patients (NISS ≥ 16) treated at Tampere University Hospital (TAUH) between 2015 and 2024. Outcomes included in-hospital mortality, prolonged hospital and ICU stay (≥ 75th percentile), in-hospital intubation, and blood transfusions. Predictive performance was assessed using the area under the receiver operating characteristic curve (AUC) and the Hosmer-Lemeshow (H-L) test for calibration. Subgroup analyses were performed for patients with significant (AIS ≥ 3) head, thorax, and extremity injuries.

**Results:**

A total of 848 (76%) patients had a higher NISS than ISS with a median increase of 9 points. Both scoring systems generally demonstrated fair to considerable (0.7 < AUC < 0.9) discrimination for mortality and intubation, and poor to fair (0.6 < AUC < 0.8) discrimination for blood transfusions and prolonged hospital/ICU stay. ISS had superior discrimination for blood transfusions in the total cohort (AUC: 0.669 vs. 0.630, *p* = 0.019), head (AUC: 0.737 vs. 0.672, *p* = 0.004), and thorax (AUC: 0.656 vs. 0.613, *p* = 0.006) subgroups, and for intubation in the head injury subgroup (AUC: 0.817 vs. 0.787 *p* = 0.038). Conversely, NISS outperformed ISS for predicting prolonged hospital stay in the extremity subgroup (AUC 0.654 vs. 0.585, *p* = 0.003).

**Conclusions:**

Contrary to its theoretical advantages, NISS does not broadly outperform ISS in severe trauma. ISS remains an equal or superior scoring system for predicting many clinical outcomes.

**Level of evidence:**

III.

**Supplementary Information:**

The online version contains supplementary material available at 10.1007/s00068-026-03198-1.

## Introduction

Trauma scoring systems are commonly used in classifying the severity of injuries. The Abbreviated Injury Scale (AIS) is widely used to rate the severity of specific injuries across the body. The AIS divides the body into nine regions, and each injury is rated on a scale from 1 (minor) to 6 (fatal).

The Injury Severity Score (ISS) was proposed in 1974 by Baker et al. [1] to evaluate overall injury severity for patients with multiple injuries. ISS divides the body into six regions: head and neck, face, thorax, abdomen, extremities, and external. Each injury is assigned an AIS score, and the ISS is calculated by summing the squares of the three highest AIS scores from three different regions with a maximum score of 75. An AIS score of 6 in any body region automatically equates to an ISS of 75.

The New Injury Severity Score (NISS) was proposed in 1997 by Osler et al. [2] as a modification to the original ISS to simplify its calculation and improve predictive power. The NISS is calculated by summing the squares of the three highest AIS scores regardless of body region. This increases total injury severity in situations where several of a patients most severe injuries are in the same body region, potentially improving outcome prediction.

While both scores were originally designed to predict mortality, their comparative utility in predicting secondary outcomes, such as prolonged hospitalization, and requirement of intubation or blood transfusions, remains a subject of ongoing debate. The validity of these scoring systems for mortality prediction has been extensively analyzed. However, the literature remains divided, with some studies reporting no significant differences while others suggest that NISS provides a marginally superior degree of accuracy [3–10]. Similarly, investigations into their predictive performance for ICU admission and length of stay have yielded conflicting results [4, 5, 9–12].

The primary aim of this study was to compare the performance of ISS and NISS in predicting clinical outcomes in severely injured trauma patients. Secondarily we compared performance in severely injured trauma patients with significant (AIS ≥ 3) head, thorax and extremity injuries.

## Methods

### Study design and patients

This retrospective cohort study used prospectively collected data from the Tampere University Hospital (TAUH) trauma registry. Injury severity was coded according to the AIS 2008 update. All registry patients are severely injured, defined as NISS ≥ 16. TAUH provides trauma care across all medical specialties and serves as the tertiary trauma care center for the surrounding 3 hospital districts, covering a population base of approximately 900 000 people.

Primarily admitted and treated patients at TAUH between 2015 and 2024 were included in this study. Patients transferred in from another hospital or transferred out early (within 48 h after admission) were excluded from this study to ensure consistent and complete availability of necessary patient data.

### Outcome measures and explanatory variables

For all patients that fit the inclusion criteria mentioned above, demographic data on ISS, NISS, injury distribution, age, sex and mechanism of injury, in-hospital mortality, length of hospital stay, ICU admission, length of ICU admission, in-hospital intubation and blood transfusions, were extracted from the registry.

The predictive performance of ISS and NISS was evaluated across five outcomes:

1. Mortality: All-cause in-hospital mortality.
2. Prolonged Hospital Stay: Defined as a length of stay ≥ 75th percentile of the total cohort distribution.
3. Prolonged ICU Stay: Defined as a length of stay ≥ 75th percentile of the total cohort distribution.
4. Intubation: Mechanical ventilation during ICU care.
5. Blood Transfusion: Administration of red blood cells or fresh frozen plasma in-hospital.

These outcomes were selected to capture both clinical severity and resource utilization. Mortality and intubation reflect critical clinical endpoints, whereas hospital/ICU length of stay and blood transfusions represent measures of resource use and injury burden.

To avoid the confounding effect of early mortality on length of stay, analysis of prolonged hospital and ICU stays was restricted to patients who survived to discharge.

### Statistical analysis

Statistical analyses were performed using R version 4.5.1 with RStudio version 2025.05.1 + 513 for Windows. Continous variables were assessed for normality using the Kolmogorov-Smirnov test [13]. Data were reported as medians with interquartile ranges (IQR) for non-normally distributed variables, and as means with standard deviations (SD) for normally distributed variables. Categorical variables were presented as absolute frequencies and percentages.

Receiver operating characteristic (ROC) curves and area under the curve (AUC) were used to assess the predictive accuracy of ISS and NISS for study outcomes. Confidence intervals for individual AUC values and the p-values for comparing the performance between the two scores were calculated using the DeLong method [14].

The following scale is used to illustrate the predictive power represented by the AUC: 0.5 < AUC < 0.6 failed prediction, 0.6 < AUC < 0.7 poor prediction, 0.7 < AUC < 0.8 fair prediction, 0.8 < AUC < 0.9 considerable, and 0.9 < AUC < 1 excellent prediction [15].

Model calibration was evaluated using the Hosmer-Lemeshow (H-L) goodness-of-fit test [16]. Patients were partitioned into deciles of predicted probability *(g =* 10) to compare observed versus expected outcomes. A p-value > 0.05 was considered to indicate acceptable model calibration.

The optimal clinical cut-off point for each score was determined using the Youden Index (*J = Sensitivity + Specificity – 1*), which represents the point of the ROC curve that maximizes the sum of sensitivity and specificity [17]. To account for uncertainty in threshold selection and diagnostic performance, 95% confidence intervals (CI) for the Youden Index, optimal cut-offs, sensitivity, and specificity were generated using non-parametric bootstrapping with 1,000 resamples [18].

A p-value of ≤ 0.05 was considered to be statistically significant.

## Results

**Fig. 1:**
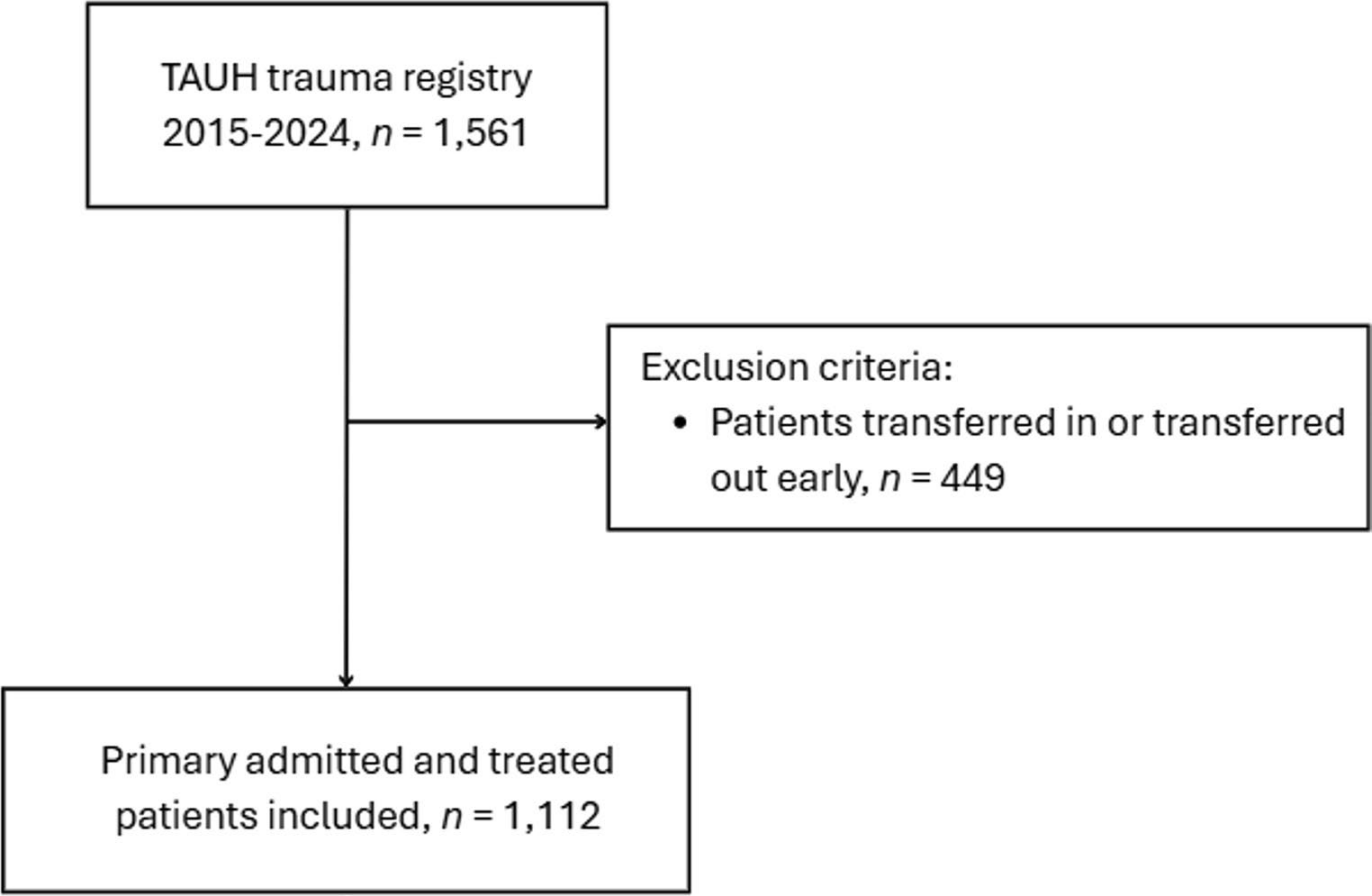
Flowchart

Between 2015 and 2024, a total of 1,561 severely injured trauma patients (NISS ≥ 16) were treated at TAUH. 449 patients were transferred in from another hospital or transferred out early (within 48 h of admission) and subsequently excluded from this study, leaving 1,112 primarily admitted and treated patients that were included (Fig. 1).

Baseline characteristics are summarized in Table 1. The median age of patients included was 50 years (IQR 28–67) and most patients were male (76%). The distribution of major injuries (AIS ≥ 3) by anatomical region was 67% for head, 38% for thorax, and 19% for extremities. Traffic accidents (45%) and falls (42%) were the primary mechanisms of injury. 42% of patients were intubated and 15% received blood transfusions. Median hospital and ICU length of stay were 7 (IQR 4–13) and 3 (IQR 1–6) days. Prolonged stays, defined by the 75th percentile of the cohort, were ≥ 13 days for hospital and ≥ 6 days for ICU stay. In-hospital mortality was 11%.

**Table 1:** Baseline characteristics of patients

	Total (*n* = 1,112)	Head AIS ≥ 3 (*n* = 746)	Thorax AIS ≥ 3 (*n* = 421)	Extremity AIS ≥ 3 (*n* = 213)
	Median (IQR)			
Age at trauma, years	50 (28–67)	55 (32–69)	44 (25–65)	39 (24–59)
ISS, score	22 (16–26)	24 (16–27)	25 (18–34)	25 (18–34)
NISS, score	27 (22–34)	29 (24–38)	29 (22–41)	27 (22–34)
NISS-ISS, score†	9 (5–13)	12 (5–16)	7 (5–9)	7 (5–9)
Hospital stay, days	7 (4–13)	7 (3–13)	8 (4–14)	11 (6–20)
ICU stay, days	3 (1–6)	3 (2–7)	3 (1–5)	3 (2–6)
	n (%)			
Sex				
Male	848 (76)	564 (76)	333 (79)	146 (69)
Female	263 (24)	182 (24)	88 (21)	67 (31)
NISS > ISS	848 (76)	564 (76)	306 (73)	131 (62)
Head AIS ≥ 3	746 (67)	746 (100)	173 (41)	68 (32)
Thorax AIS ≥ 3	421 (38)	173 (23)	421 (100)	110 (52)
Extremity AIS ≥ 3	213 (19)	68 (9)	110 (26)	213 (100)
ICU admission	1091 (98)	731 (98)	416 (99)	207 (97)
Intubation in-hospital	470 (42)	370 (50)	185 (44)	100 (47)
Blood transfusion	165 (15)	83 (11)	87 (21)	76 (36)
In-hospital mortality	124 (11)	110 (15)	41 (10)	21 (10)
Mechanism of injury				
Fall	470 (42)	385 (52)	104 (25)	50 (24)
Traffic	504 (45)	267 (36)	277 (66)	146 (69)
Other	138 (13)	94 (12)	40 (10)	17 (8)

IQR: interquartile range, NISS: new injury severity scale, ISS: injury severity scale, ICU: intensive care unit, AIS: abbreviated injury scale

Continuous variables are reported as medians and interquartile ranges, as all were found to be non-normally distributed via the Kolmogorov-Smirnov test

†NISS-ISS calculated only for patients with NISS > ISS

**Fig. 2:**
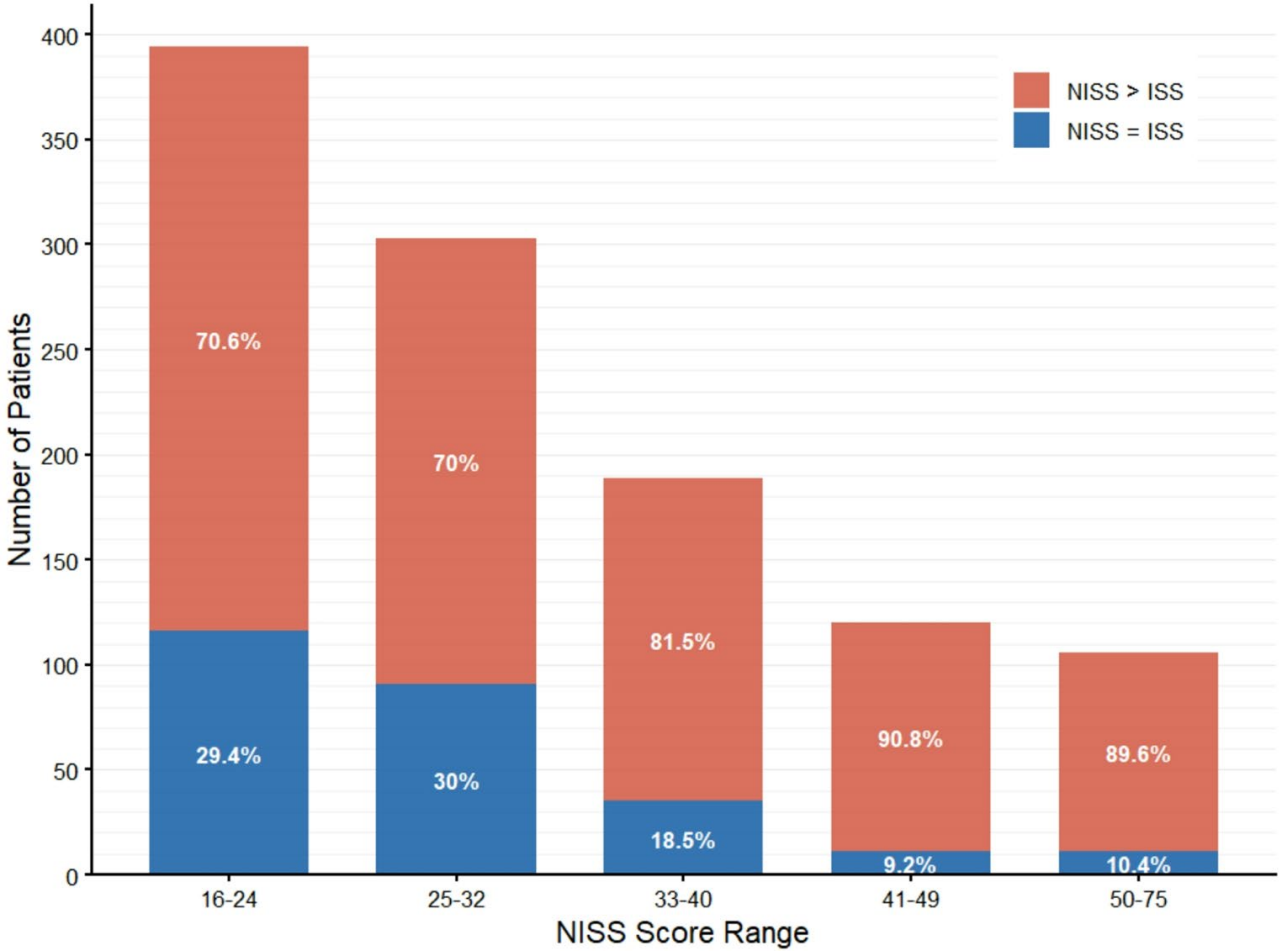
Distribution of NISS reclassification by injury severity

The median ISS and NISS for the total cohort were 22 (IQR 16–26) and 27 (IQR 22–34), respectively. NISS reclassified 848 patients (76%) to a higher score than the ISS, with a median increase of 9 points (IQR 5–13) among those reclassified. Patients with major head injuries had the highest rate of reclassification (76%) and the largest median score increase of 12 (IQR 5–16). In contrast, patients with major extremity injuries had the lowest reclassification rate (62%). Overall, reclassification frequency increased in tandem with total injury severity (Fig. 2).

### Predictive performance: total cohort

Table 2 summarizes the discrimination and calibration of ISS and NISS for the total cohort (*n* = 1,112). Both scores demonstrated their highest discriminative power in predicting in-hospital mortality (ISS AUC: 0.752, NISS AUC: 0.777) and intubation (ISS AUC: 0.774, NISS AUC: 0.767). Both scoring systems had poor discrimination for prolonged hospital (ISS AUC: 0.654, NISS AUC: 0.663) and ICU stay (ISS AUC: 0.682, NISS AUC: 0.698). ISS showed significantly better discrimination than NISS in predicting blood transfusions (AUC 0.668 vs. 0.630, *p* = 0.019), although both scores performed poorly.

**Table 2:** Comparative Performance of ISS and NISS in Predicting Clinical Outcomes for Whole Cohort (*n* = 1112)

Outcome	ISS		NISS		*p*-value†
	AUC (95% CI)	H-L (*p*-value)	AUC (95% CI)	H-L (*p*-value)	
In-hospital mortality	0.752 (0.712–0.792)	25.99 (< 0.001)	0.777 (0.734–0.819)	34.98 (< 0.001)	0.209
Prolonged hospital stay	0.654 (0.616–0.691)	3.37 (0.339)	0.663 (0.626–0.699)	8.61 (0.035)	0.543
Prolonged ICU stay	0.682 (0.643–0.720)	2.16 (0.540)	0.698 (0.660–0.735)	3.66 (0.300)	0.291
In-hospital intubation	0.774 (0.746–0.801)	12.22 (0.007)	0.767 (0.740–0.795)	21.30 (< 0.001)	0.589
Blood transfusions	0.669 (0.625–0.713)	9.03 (0.029)	0.630 (0.586–0.675)	2.93 (0.403)	**0.019**

AUC: area under the receiver operating characteristic curve, CI: confidence interval, H-L: Hosmer–Lemeshow test, ISS: Injury Severity Score, NISS: New Injury Severity Score, ICU: intensive care unit

H-L values represent the chi-square statistic; *p* > 0.05 indicates a good fit between predicted and observed outcomes

Prolonged hospital stay and ICU stay calculated from survivors (*n* = 988)

† Statistical comparison of AUCs performed with DeLong’s test

ISS showed acceptable calibration in predicting prolonged hospital (*p* = 0.339) and ICU stay (*p* = 0.540) while NISS showed acceptable calibration in predicting prolonged ICU stay (*p* = 0.300) and blood transfusions (*p* = 0.403).

### Predictive performance: subgroup analysis

In patients with head injuries, ISS demonstrated significantly better discrimination for both intubation (AUC 0.817 vs. 0.787, *p* = 0.038) and blood transfusions (AUC 0.737 vs. 0.672, *p* = 0.004). ISS showed acceptable calibration for prolonged hospital and ICU stays, while NISS showed acceptable calibration for prolonged ICU stay and blood transfusions (Table 3).

**Table 3:** Comparative Performance of ISS and NISS in Predicting Clinical Outcomes for Patients with Head Injuries (*n* = 746)

Outcome	ISS		NISS		*p*-value†
	AUC (95% CI)	H-L (*p*-value)	AUC (95% CI)	H-L (*p*-value)	
In-hospital mortality	0.724 (0.679–0.769)	34.72 (< 0.001)	0.753 (0.704–0.802)	15.49 (< 0.001)	0.202
Prolonged hospital stay	0.680 (0.635–0.725)	4.71 (0.194)	0.677 (0.633–0.722)	9.03 (0.029)	0.870
Prolonged ICU stay	0.672 (0.628–0.716)	1.87 (0.600)	0.668 (0.624–0.712)	2.93 (0.403)	0.827
In-hospital intubation	0.817 (0.787–0.847)	11.67 (0.009)	0.787 (0.756–0.819)	12.13 (0.007)	**0.038**
Blood transfusions	0.737 (0.683–0.792)	16.08 (< 0.001)	0.672 (0.613–0.713)	2.78 (0.427)	**0.004**

AUC: area under the receiver operating characteristic curve, CI: confidence interval, H-L: Hosmer–Lemeshow test, ISS: Injury Severity Score, NISS: New Injury Severity Score, ICU: intensive care unit

H-L values represent the chi-square statistic; *p* > 0.05 indicates a good fit between predicted and observed outcomes

Prolonged hospital stay and ICU stay calculated from survivors (*n* = 636)

† Statistical comparison of AUCs performed with DeLong’s test

In patients with thorax injuries, ISS demonstrated significantly better discrimination for blood transfusions (AUC 0.656 vs. 0.613, *p* = 0.006). NISS showed acceptable calibration across all outcomes (*p* > 0.05). In contrast, ISS failed to reach acceptable calibration for blood transfusions (*p* = 0.003), though it remained acceptable for all other outcomes (Table 4).

**Table 4:** Comparative performance of ISS and NISS in predicting clinical outcomes for patients with thorax injuries (*n* = 421)

Outcome	ISS		NISS		*p*-value†
	AUC (95% CI)	H-L (*p*-value)	AUC (95% CI)	H-L (*p*-value)	
In-hospital mortality	0.810 (0.741–0.880)	2.83 (0.419)	0.841 (0.778–0.904)	1.69 (0.639)	0.182
Prolonged hospital stay	0.699 (0.645–0.753)	5.44 (0.142)	0.681 (0.625–0.736)	1.38 (0.709)	0.189
Prolonged ICU stay	0.766 (0.713–0.819)	5.30 (0.151)	0.748 (0.694–0.803)	3.55 (0.315)	0.212
In-hospital intubation	0.792 (0.749–0.835)	0.88 (0.813)	0.777 (0.732–0.812)	1.66 (0.646)	0.212
Blood transfusions	0.656 (0.598–0.715)	14.15 (0.003)	0.613 (0.550–0.675)	2.64 (0.451)	**0.006**

AUC: area under the receiver operating characteristic curve, CI: confidence interval, H-L: Hosmer–Lemeshow test, ISS: Injury Severity Score, NISS: New Injury Severity Score, ICU: intensive care unit

H-L values represent the chi-square statistic; *p* > 0.05 indicates a good fit between predicted and observed outcomes

Prolonged hospital stay and ICU stay calculated from survivors (*n* = 380)

† Statistical comparison of AUCs performed with DeLong’s test

In patients with extremity injuries, NISS demonstrated significantly better discrimination for prolonged hospital stay (AUC 0.654 vs. 0.585, *p* = 0.003). ISS showed acceptable calibration across all outcomes (*p* > 0.05). Conversely, NISS failed to demonstrate acceptable calibration in predicting intubation (*p* = 0.037) but was acceptable for all other parameters (Table 5).

**Table 5:** Comparative performance of ISS and NISS in predicting clinical outcomes for patients with extremity injuries (*n* = 213)

Outcome	ISS		NISS		*p*-value†
	AUC (95% CI)	H-L (*p*-value)	AUC (95% CI)	H-L (*p*-value)	
In-hospital mortality	0.807 (0.720–0.894)	4.22 (0.239)	0.810 (0.722–0.899)	0.62 (0.101)	0.875
Prolonged hospital stay	0.585 (0.504–0.667)	6.95 (0.073)	0.654 (0.578–0.730)	1.50 (0.472)	**0.003**
Prolonged ICU stay	0.757 (0.678–0.835)	2.03 (0.567)	0.756 (0.684–0.828)	0.75 (0.687)	0.971
In-hospital intubation	0.714 (0.644–0.783)	1.82 (0.611)	0.728 (0.662–0.794)	8.49 (0.037)	0.482
Blood transfusions	0.578 (0.499–0.656)	1.73 (0.630)	0.593 (0.516–0.670)	1.91 (0.592)	0.466

AUC: area under the receiver operating characteristic curve, CI: confidence interval, H-L: Hosmer–Lemeshow test, ISS: Injury Severity Score, NISS: New Injury Severity Score, ICU: intensive care unit

H-L values represent the chi-square statistic; *p* > 0.05 indicates a good fit between predicted and observed outcomes

Prolonged hospital stay and ICU stay calculated from survivors (*n* = 192)

† Statistical comparison of AUCs performed with DeLong’s test

**Fig. 3:**
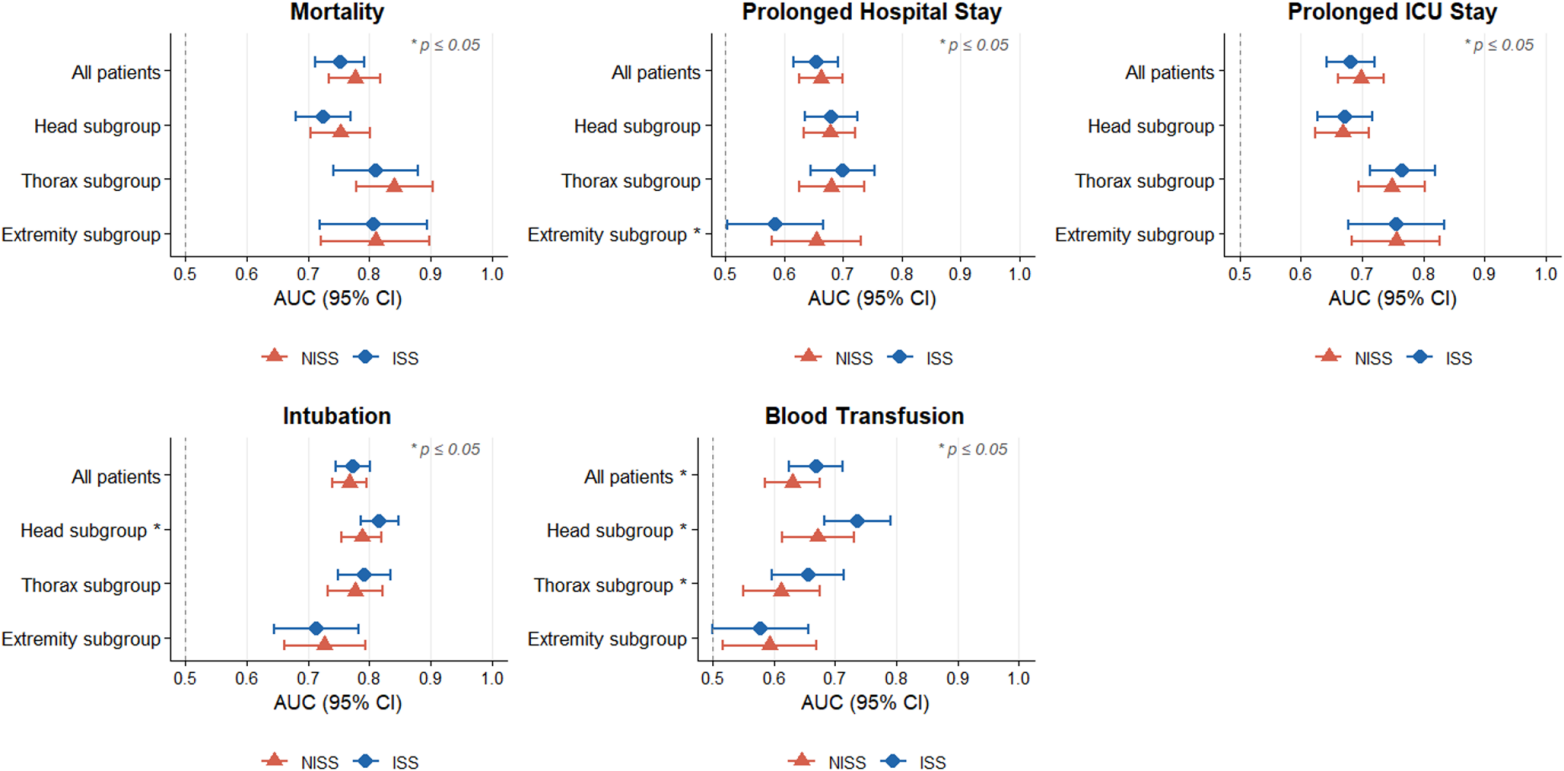
Area under the receiver operating characteristic curve (ROC - AUC) analysis for ISS and NISS by outcome and subgroup

Figure 3. summarizes predictive performance (AUC) for all outcomes and subgroups. Supplementary Tables 1–4 contain optimal cut-offs and associated Youden indices, sensitivity and specificity for the whole cohort and subgroups. Supplementary Figs. 1–4 depict ROC curves for each outcome.

## Discussion

The present study provides a comprehensive evaluation of the Injury Severity Score (ISS) and the New Injury Severity Score (NISS) in predicting clinical outcomes and resource utilization across a cohort of 1,112 severely injured trauma patients (NISS ≥ 16). Most patients demonstrated discordant severity scores (NISS > ISS), with NISS reclassifying 76% of patients to a higher severity score. This upward shift in predicted risk did not consistently translate into improved discriminative performance. To our knowledge, few studies have compared the predictive performance of NISS and ISS for clinical outcomes in a cohort restricted to severely injured trauma patients [8].

### In-hospital mortality

For in-hospital mortality, no statistically significant differences in discriminative power were observed between the two scoring systems in the whole cohort or any specific subgroup. Both scores reached their highest AUC values for mortality in the thorax and extremity subgroups (AUC > 0.80).

Earlier studies have indicated that NISS has similar or better predictive value for mortality in trauma patients. Large-scale registry studies by Lavoie et al. [19] and Harwood et al. [4] found that NISS demonstrated better discrimination predicting mortality in trauma patients. In contrast, several smaller scale studies have found no statistically significant difference between NISS and ISS in predicting mortality [3, 5, 7, 8]. This suggests that while large-scale registry studies can detect the marginal statistical benefit of NISS over ISS, this difference may be too subtle to be captured in smaller-scale studies or to provide a meaningful advantage in routine clinical survival benchmarking.

### Hospital and ICU length of stay

NISS demonstrated a significant discriminative advantage for prolonged hospital stay in the extremity subgroup (AUC 0.654 vs. 0.585, *p* = 0.003). This likely reflects the cumulative surgical and rehabilitative burden associated with multiple orthopedic injuries [20, 21]. Unlike the ISS, which limits the contribution of a single anatomical region, the NISS aggregates multiple severe injuries within the same region, effectively capturing the increased resource utilization associated with complex orthopedic trauma.

Conversely, both scores showed fair and comparable prediction for prolonged ICU stay. Notably, both systems showed better discrimination for prolonged ICU stay than prolonged hospital stay in all groups, indicating that overall injury severity is more related to ICU stay than total hospital stay.

Prior literature is discrepant on the predictive power of NISS and ISS on prolonged hospital and ICU stay. Our findings align with studies by Harwood et al. [4], Lavoie at al. [12] and Ede et al. [10] which found that NISS demonstrated better discrimination for predicting prolonged hospital stay. Conversely an earlier study by Tamim et al. [5] found ISS to better predict prolonged hospital stay than NISS in trauma patients. Some studies have also found NISS to be a better predictor for prolonged ICU stay than ISS [4, 8].

### Blood transfusions and intubation

A significant finding of this study is the superior discriminative performance of ISS over NISS in predicting blood transfusions across the total cohort (AUC: 0.669 vs. 0.630, *p* = 0.019), as well as in the head (AUC: 0.737 vs. 0.672, *p* = 0.004) and thorax (AUC: 0.656 vs. 0.613, *p* = 0.006) subgroups. This finding suggests that anatomic diversity required by the ISS calculation may serve as a superior proxy for the requirement of blood transfusions. We found that ISS also demonstrates better discrimination for intubation in patients with head injuries (AUC: 0.817 vs. 0.787 *p* = 0.038). This is likely due to head injury severity being the most important driver for requiring intubation [22]. While the NISS captures localized injury density, which reclassifies most patients to a higher injury severity, this increased severity does not translate to higher rates of blood transfusions or intubation.

Our findings differ from an earlier study which found no significant difference between discrimination for blood transfusions in patients with musculoskeletal injuries between NISS and ISS [10]. No prior study has analyzed the discriminative ability of NISS and ISS in predicting intubation in patients with head injuries, making this a novel finding of our study. A previous study by Jin et al. [7] found no significant difference between scoring systems in predicting intubation in patients with thoracic trauma and a study by Honarmand et al. [23] found NISS to demonstrate better discrimination in trauma patients.

### Model calibration and stability

While both scores exhibited moderate discrimination in the whole cohort, both also failed calibration for multiple outcomes. This suggests that while both scoring systems are effective in ranking patients by risk, they lack the precision required to accurately predict absolute risk. This finding was also observed in the head trauma subgroup. In contrast, both scores showed acceptable calibration in thorax and extremity injury subgroups for most outcomes.

Calibration estimated by the Hosmer-Lemeshow (H-L) test should be interpreted with caution, especially when comparing calibration between different subgroups and studies with different sample sizes. The H-L test is known to be sensitive to large sample sizes, often yielding significant p-values for clinically negligible deviations [24]. This statistical phenomenon can partially explain the finding of both scoring systems failing calibration in the whole cohort for multiple outcomes but having acceptable calibration for most outcomes in the smaller thorax and extremity subgroups.

Beyond sample size, the improved calibration in these subgroups likely reflects the homogeneity of patients with specific injury patterns. Severely injured trauma patients form a diverse cohort where identical severity scores can represent vastly different physiological trajectories. By isolating specific injury patterns, we evaluate a more clinically uniform population where the relationship between trauma scores and outcomes becomes more consistent. In these homogenous groups, anatomic scores align more closely with the actual clinical course, allowing them to function as more reliable indicators of absolute risk.

### Strengths and limitations

This study has several limitations. Its retrospective, registry-based nature meant that analyses were constrained by the availability and accuracy of recorded data. Additionally, the single-center setting may limit the generalizability of our findings to other trauma systems that differ in patient populations, management protocols, or available resources.

Despite these limitations, the study has notable strengths. The cohort was prospectively collected, representing a comprehensive sample of severely injured patients treated at a tertiary trauma center. We had access to comprehensive data for the whole cohort and a sample size which enabled subgroup analysis.

## Conclusion

Contrary to the theoretical advantage of NISS, our study demonstrates that ISS remains a superior or equal predictor for many clinical outcomes. ISS demonstrated better predictive power for blood transfusions in the whole cohort as well as head and thorax injury subgroups. ISS also outperformed NISS in predicting intubation in patients with head trauma. On the contrary, NISS showed better discrimination for prolonged hospital stay in patients with extremity injuries. Both systems exhibited calibration limitations in the larger cohort, indicating that their absolute risk predictions should be applied with caution in heterogeneous populations.

These findings may serve as a reference for the clinical and scientific application of these two trauma severity scoring systems in evaluating injury and predicting clinical outcomes and complications.

## Electronic Supplementary Material

Below is the link to the electronic supplementary material.

Supplementary Material 1

## Data Availability

Data and materials available from the corresponding author on reasonable request.

## Abbreviations

TAUH: Tampere University Hospital
AIS: Abbreviated Injury Scale
ISS: Injury Severity Score
NISS: New Injury Severity Score
ICU: Intensive Care Unit
GCS: Glasgow Coma Scale
IQR: Interquartile Range
CI: Confidence Interval
AUC: Area Under the Curve
ROC: Receiver Operating Characteristic
H-L: Hosmer-Lemeshow
