# Supplementary material for: Predictive performance of ISS and NISS for clinical outcomes in severely injured trauma patients: a retrospective registry study
Source: Eur J Trauma Emerg Surg. 2026 Apr 28;52(1):152. doi: 10.1007/s00068-026-03198-1 (PMC13124834; doi:10.1007/s00068-026-03198-1)

| Supplementary Table 1. Optimal cut-off values and diagnostic performance metrics for ISS and NISS in the total cohort (n=1112). | | | | |
| --- | --- | --- | --- | --- |
|  | Cutoff (95% CI) | Sensitivity (95% CI) | Specificity (95% CI) | Youden index (95% CI) |
| In-hospital mortality |  |  |  |  |
| ISS | 25 (23-25) | 0.879 (0.827-0.936) | 0.633 (0.596-0.662) | 0.512 (0.450-0.570) |
| NISS | 33 (25-44) | 0.734 (0.471-0.962) | 0.672 (0.397-0.935) | 0.406 (0.342-0.494) |
| Prolonged hospital stay |  |  |  |  |
| ISS | 18 (18-26) | 0.729 (0.409-0.773) | 0.506 (0.483-0.820) | 0.235 (0.189-0.308) |
| NISS | 27 (25-34) | 0.732 (0.466-0.796) | 0.521 (0.470-0.785) | 0.253 (0.207-0.324) |
| Prolonged ICU stay |  |  |  |  |
| ISS | 25 (20-27) | 0.586 (0.427-0.719) | 0.712 (0.581-0.854) | 0.297 (0.232-0.362) |
| NISS | 30 (25-34) | 0.563 (0.512-0.859) | 0.738 (0.453-0.781) | 0.301 (0.254-0.371) |
| In-hospital intubation |  |  |  |  |
| ISS | 25 (20-25) | 0.687 (0.663-0.836) | 0.768 (0.623-0.796) | 0.455 (0.416-0.515) |
| NISS | 30 (25-33) | 0.619 (0.584-0.880) | 0.738 (0.509-0.817) | 0.403 (0.358-0.465) |
| Blood transfusions |  |  |  |  |
| ISS | 26 (18-27) | 0.533 (0.445-0.831) | 0.744 (0.448-0.811) | 0.278 (0.211-0.358) |
| NISS | 31 (26-40) | 0.552 (0.373-0.808) | 0.655 (0.420-0.821) | 0.206 (0.142-0.284) |
| CI: confidence interval, ISS: Injury Severity Score, NISS: New Injury Severity Score, ICU: intensive care unit | | | | |
| Prolonged hospital stay and ICU stay calculated from survivors (n=988) | | |  |  |

| Supplementary Table 2. Optimal cut-off values and diagnostic performance metrics for ISS and NISS in the head subgroup (n=746). | | | | |
| --- | --- | --- | --- | --- |
|  | Cutoff (95% CI) | Sensitivity (95% CI) | Specificity (95% CI) | Youden index (95% CI) |
| In-hospital mortality |  |  |  |  |
| ISS | 25 (25-25) | 0.900 (0.838-0.951) | 0.571 (0.531-0.607) | 0.471 (0.401-0.533) |
| NISS | 35 (25-49) | 0.609 (0.417-0.952) | 0.763 (0.359-0.931) | 0.372 (0.300-0.472) |
| Prolonged hospital stay |  |  |  |  |
| ISS | 18 (18-27) | 0.799 (0.472-0.855) | 0.493 (0.454-0.822) | 0.292 (0.228-0.370) |
| NISS | 33 (25-34) | 0.571 (0.520-0.885) | 0.706 (0.392-0.7539 | 0.276 (0.215-0.363) |
| Prolonged ICU stay |  |  |  |  |
| ISS | 25 (19-28) | 0.596 (0.377-0.762) | 0.661 (0.523-0.875) | 0.257 (0.211-0.349) |
| NISS | 30 (25-34) | 0.565 (0.500-0.857) | 0.702 (0.407-0.766) | 0.267 (0.209-0.353) |
| In-hospital intubation |  |  |  |  |
| ISS | 21 (20-25) | 0.846 (0.745-0.893) | 0.678 (0.623-0.790) | 0.524 (0.476-0.591) |
| NISS | 30 (28-33) | 0.668 (0.621-0.770) | 0.779 (0.675-0.825) | 0.447 (0.385-0.512) |
| Blood transfusions |  |  |  |  |
| ISS | 26 (26-27) | 0.711 (0.580-0.840) | 0.710 (0.665-0.795) | 0.421 (0.327-0.521) |
| NISS | 34 (27-40) | 0.675 (0.454-0.877) | 0.618 (0.397-0.800) | 0.293 (0.200-0.401) |
| CI: confidence interval, ISS: Injury Severity Score, NISS: New Injury Severity Score, ICU: intensive care unit | | | | |
| Prolonged hospital stay and ICU stay calculated from survivors (n=636) | | |  |  |

| Supplementary Table 3. Optimal cut-off values and diagnostic performance metrics for ISS and NISS in the head subgroup (n=421). | | | | |
| --- | --- | --- | --- | --- |
|  | Cutoff (95% CI) | Sensitivity (95% CI) | Specificity (95% CI) | Youden index (95% CI) |
| In-hospital mortality |  |  |  |  |
| ISS | 34 (25-37) | 0.780 (0.641-0.947) | 0.753 (0.546-0.872) | 0.533 (0.417-0.676) |
| NISS | 40 (31-49) | 0.780 (0.600-0.974) | 0.782 (0.564-0.926) | 0.562 (0.463-0.702) |
| Prolonged hospital stay |  |  |  |  |
| ISS | 27 (22-28) | 0.620 (0.527-0.832) | 0.701 (0.472-0.784) | 0.321 (0.230-0.425) |
| NISS | 34 (26-34) | 0.605 (0.515-0.876) | 0.685 (0.403-0.763) | 0.290 (0.198-0.396) |
| Prolonged ICU stay |  |  |  |  |
| ISS | 27 (25-34) | 0.722 (0.535-0.848) | 0.700 (0.598-0.872) | 0.421 (0.347-0.535) |
| NISS | 34 (32-37) | 0.711 (0.526-0.815) | 0.689 (0.632-0.857) | 0.400 (0.309-0.508) |
| In-hospital intubation |  |  |  |  |
| ISS | 27 (25-34) | 0.697 (0.515-0.821) | 0.737 (0.626-0.915) | 0.435 (0.374-0.530) |
| NISS | 34 (31-40) | 0.703 (0.480-0.767) | 0.729 (0.675-0.920) | 0.432 (0.354-0.515) |
| Blood transfusions |  |  |  |  |
| ISS | 27 (19-28) | 0.678 (0.574-0.945) | 0.605 (0.311-0.688) | 0.283 (0.192-0.400) |
| NISS | 32 (19-40) | 0.632 (0.417-0.989) | 0.572 (0.148-0.765) | 0.204 (0.119-0.319) |
| CI: confidence interval, ISS: Injury Severity Score, NISS: New Injury Severity Score, ICU: intensive care unit | | | | |
| Prolonged hospital stay and ICU stay calculated from survivors (n=380) | | |  |  |

| Supplementary Table 4. Optimal cut-off values and diagnostic performance metrics for ISS and NISS in the extremity subgroup (n=213). | | | | |
| --- | --- | --- | --- | --- |
|  | Cutoff (95% CI) | Sensitivity (95% CI) | Specificity (95% CI) | Youden index (95% CI) |
| In-hospital mortality |  |  |  |  |
| ISS | 31 (23-34) | 0.762 (0.667-1) | 0.734 (0.485-0.808) | 0.496 (0.394-0.667) |
| NISS | 32 (32-49) | 0.857 (0.592-1) | 0.656 (0.588-0.944) | 0.513 (0.353-0.673) |
| Prolonged hospital stay |  |  |  |  |
| ISS | 28 (24-31) | 0.429 (0.355-0.624) | 0.787 (0.592-0.860) | 0.216 (0.109-0.354) |
| NISS | 27 (26-32) | 0.786 (0.409-0.859) | 0.474 (0.408-0.839) | 0.264 (0.161-0.399) |
| Prolonged ICU stay |  |  |  |  |
| ISS | 27 (27-32) | 0.727 (0.528-0.839) | 0.723 (0.655-0.892) | 0.450 (0.337-0.602) |
| NISS | 32 (26-32) | 0.636 (0.500-0.946) | 0.774 (0.436-0.854) | 0.410 (0.285-0.548) |
| In-hospital intubation |  |  |  |  |
| ISS | 31 (22-32) | 0.500 (0.430-0.818) | 0.850 (0.535-0.905) | 0.350 (0.259-0.484) |
| NISS | 32 (25-32) | 0.590 (0.486-0.888) | 0.779 (0.440-0.865) | 0.369 (0.262-0.495) |
| Blood transfusions |  |  |  |  |
| ISS | 22 (17-36) | 0.750 (0.411-0.944) | 0.431 (0.164-0.759) | 0.181 (0.065-0.320) |
| NISS | 27 (21-36) | 0.789 (0.318-0.969) | 0.372 (0.152-0.839) | 0.162 (0.08-0.303) |
| CI: confidence interval, ISS: Injury Severity Score, NISS: New Injury Severity Score, ICU: intensive care unit | | | | |
| Prolonged hospital stay and ICU stay calculated from survivors (n=192) | | |  |  |

Supplementary Figure 1. Comparison of ROC curves between ISS and NISS for all outcomes in the total cohort (n=1,112).
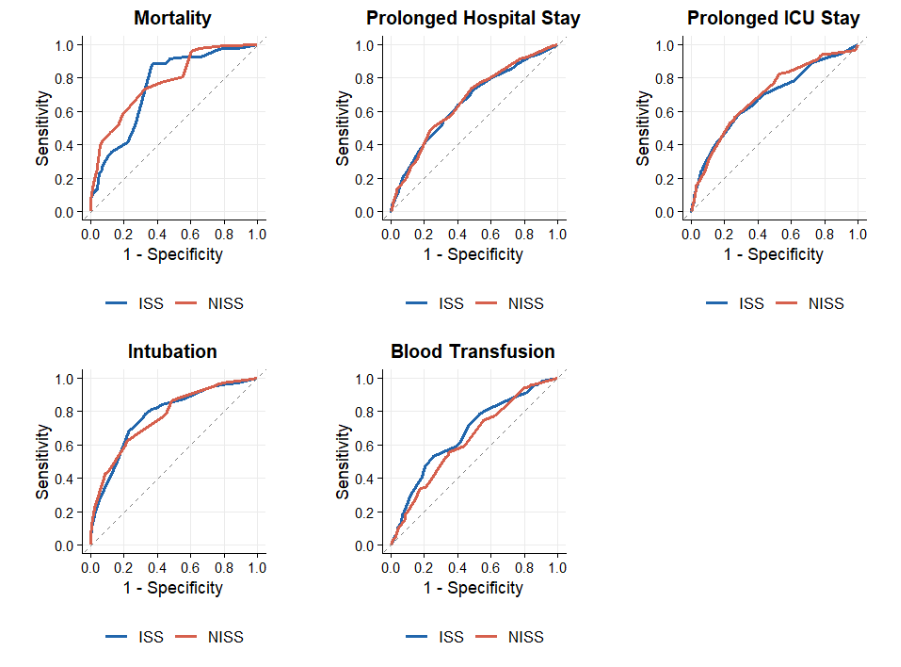


Supplementary Figure 2. Comparison of ROC curves between ISS and NISS for all outcomes in the head injury subgroup (n=746).


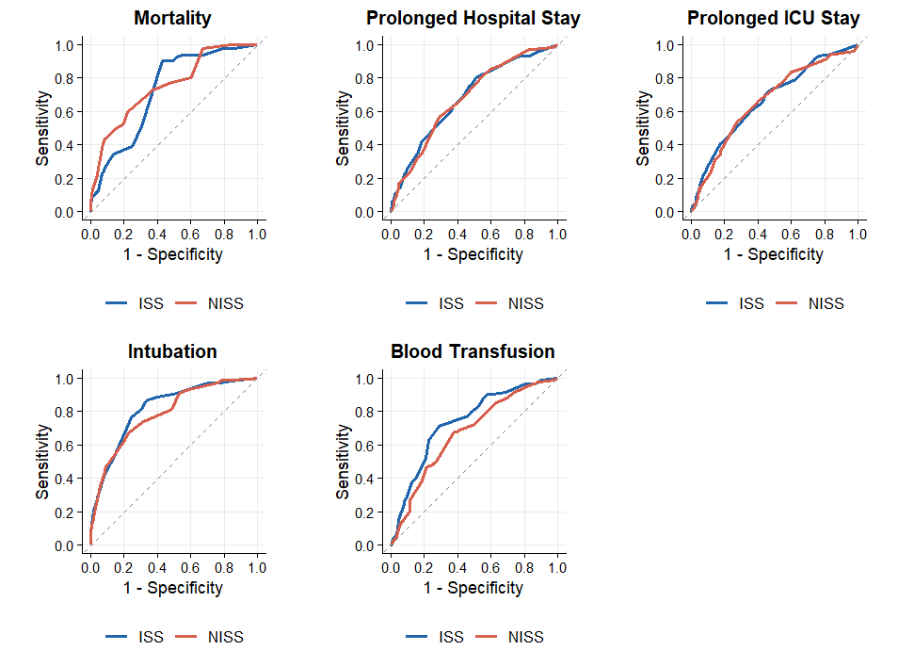


Supplementary Figure 3. Comparison of ROC curves between ISS and NISS for all outcomes in the thorax injury subgroup (n=421).


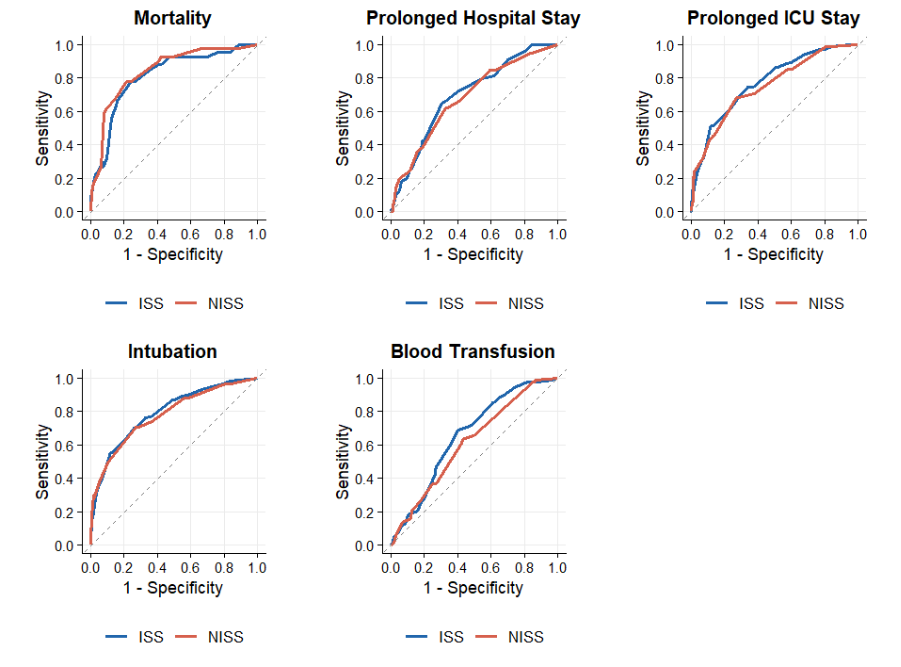


Supplementary Figure 4. Comparison of ROC curves between ISS and NISS for all outcomes in the extremity injury subgroup (n=213).


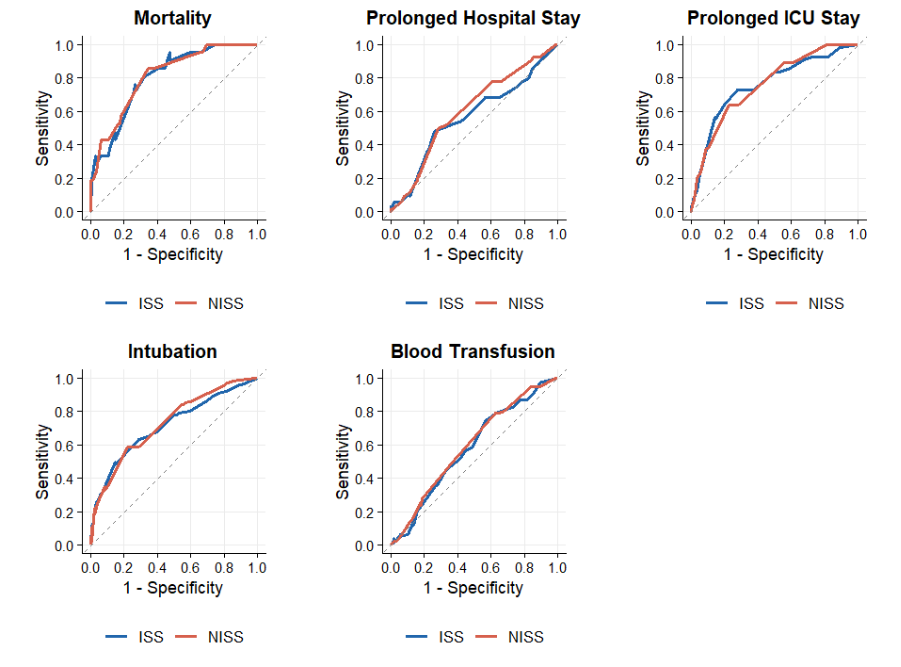

Supplement: Supplementary file 1 — Supplementary Material 1 [file 68_2026_3198_MOESM1_ESM.docx]
